# Supramolecular Photosensitizers Based on HMeQ[6] and Their Photodynamic Effects on Triple-Negative Breast Cancer Cells

**DOI:** 10.3390/molecules30234576

**Published:** 2025-11-28

**Authors:** Beibei Song, Qingyi Kong, Bo Xiao, Ting Huang, Yan Su, Baofei Sun, Guangwei Feng, Xiaojun Wen, Jian Feng

**Affiliations:** Key Laboratory of Human Brain Bank for Functions and Diseases, Guizhou Provincial Department of Education, School of Basic Medical Sciences, Guizhou Medical University, Guiyang 550025, China; song2401519@163.com (B.S.); kqy18313841355@163.com (Q.K.); 16687816631@163.com (T.H.);

**Keywords:** supramolecular photosensitizer, cucurbit[6]uril, porphyrin, 4T1 cell

## Abstract

The principal challenge in the development of efficient porphyrin-based photosensitizers is the intrinsic aggregation-induced quenching effect, which significantly impairs the generation efficiency of singlet oxygen (^1^O_2_) in photodynamic therapy (PDT). This study addresses this limitation through a supramolecular approach grounded in host-guest chemistry. Partially methyl-substituted cucurbit[6]uril (HMeQ[6]) was selected as the macrocyclic host owing to its smaller portal size and larger outer diameter, features that facilitate both strong binding affinity and effective spatial isolation. A porphyrin derivative functionalized with two cationic arms (DPPY) was designed and synthesized as the guest molecule. The results derived from ^1^H NMR titration and UV spectroscopy analyses demonstrate that, in aqueous solution, these components self-assemble via host-guest interactions to form a 2:1 stoichiometric supramolecular complex (DPPY@HMeQ[6]) with a binding constant of 2.11 × 10^5^ M^−1^. TEM, AFM, and DLS analyses indicate that this complex further assembles into nanosheet structures with dimensions of approximately 100 nm. Spectroscopic analyses reveal that encapsulation by HMeQ[6] effectively inhibits π-π stacking aggregation of DPPY molecules, resulting in an approximate threefold increase in fluorescence intensity and an extension of fluorescence lifetime from 3.2 ns to 6.2 ns. Relative to free DPPY, the complex demonstrates a sixfold enhancement in ^1^O_2_ generation efficiency. Subsequently, 4T1 cells, derived from mouse triple-negative breast tumors, were selected as the experimental model. These cells exhibit high invasiveness and metastatic potential, thereby effectively recapitulating the pathological progression of human triple-negative breast cancer. In vitro cellular assays indicate efficient internalization of the complex by 4T1 cells, inducing a concentration-dependent increase in reactive oxygen species (ROS) and oxidative stress following light irradiation. The in vitro cytotoxicity of the supramolecular photosensitizer was assessed employing the CCK-8 assay and flow cytometry techniques. The half-maximal inhibitory concentration (IC_50_) against cancer cells is 1.8 μM, with apoptosis rates reaching up to 65.3%, while exhibiting minimal dark toxicity. This study expands the potential applications of methyl-substituted cucurbiturils within functional supramolecular assemblies and proposes a viable approach for the development of efficient and activatable supramolecular photosensitizers.

## 1. Introduction

Photodynamic Therapy (PDT) represents a non-invasive approach in cancer treatment, fundamentally relying on the excitation of photosensitizers (PSs) by light at specific wavelengths. This excitation facilitates energy transfer to adjacent oxygen molecules, producing highly reactive oxygen species (ROS), particularly singlet oxygen (^1^O_2_), which subsequently induces apoptosis or necrosis in tumor cells [1,2,3]. Compared to conventional treatment modalities, PDT offers several distinct advantages. The generation of ROS is contingent upon light activation, thereby enabling precise spatial and temporal control over cytotoxic effects [4,5]. Moreover, ROS-mediated cytotoxicity is associated with minimal adverse effects, a reduced propensity for drug resistance development, and the capacity to stimulate anti-tumor immune responses, underscoring its considerable potential in oncological therapy [6,7]. Despite these advantages, PDT applications continue to encounter several fundamental challenges. First, traditional photosensitizers are hindered by material science limitations, including complex synthesis processes, poor water solubility, and inadequate targeting capabilities. Second, the penetration depth of light in tissue is constrained by its wavelength; within the optimal therapeutic window (650–900 nm), achieving both deep tissue penetration and efficient singlet oxygen generation remains problematic. Third, the hypoxic nature of the tumor microenvironment, combined with the short lifespan and limited diffusion distance of reactive oxygen species, further diminishes therapeutic efficacy [8,9]. Among various PSs, porphyrins and their derivatives are extensively employed in PDT due to their superior light-harvesting abilities and favorable redox characteristics, which facilitate efficient ^1^O_2_ production upon photoexcitation [10,11]. Nonetheless, the planar macrocyclic structure of porphyrins predisposes them to strong π–π stacking interactions, resulting in aggregation in solution. This aggregation promotes non-radiative decay pathways that dissipate excited-state energy and markedly reduce the lifetime of charge-separated states, thereby diminishing fluorescence quantum yield and ROS generation efficiency—a phenomenon termed aggregation-caused quenching (ACQ) [12,13]. Consequently, the development of highly efficient porphyrin-based photosensitizers capable of mitigating the ACQ effect is critical for improving the therapeutic efficacy of PDT.

To date, various molecular engineering strategies have been developed to inhibit the aggregation of porphyrin molecules by spatially separating them: (1) dispersing or encapsulating porphyrin molecules within inorganic or organic matrices [14,15]; (2) synthesizing dendritic molecules centered on porphyrin cores [16,17]; and (3) introducing bulky substituents around the porphyrin periphery [18,19]. Although these innovative approaches effectively prevent π–π stacking of the porphyrin core, challenges remain due to the complexity of synthetic procedures and the instability of the resulting nanostructures. In contrast, supramolecular engineering strategies based on host–guest chemistry provide a straightforward means to address these issues [20]. By encapsulating porphyrin derivatives through host–guest interactions with supramolecular macrocycles—such as cyclodextrins, cucurbiturils, and pillararenes—their aggregation can be substantially suppressed. This suppression mitigates ACQ, enhances ROS generation, and improves the therapeutic efficacy of PDT [21,22,23]. For instance, Liu et al. developed an amphiphilic fluorinated β-cyclodextrin assembly whose fluorinated structure effectively enriches oxygen and alleviates tumor hypoxia, while simultaneously inhibiting photosensitizer aggregation and significantly enhancing singlet oxygen generation efficiency [24]. Similarly, Zhang et al. employed pillararenes to construct a supramolecular nanoplatform that, via pH-responsive reversible host–guest interactions, selectively dissociates and activates photosensitizing components within the acidic tumor microenvironment, resulting in a 5.6-fold increase in singlet oxygen yield compared to neutral conditions [25]. Furthermore, cucurbituril-based polymer vesicles can spatially arrange porphyrins on their surfaces at defined intervals, thereby augmenting singlet oxygen production [26,27]. Despite these advances, the complex tumor microenvironment—characterized by abnormal pH, enzymatic activity, and competitive molecular species—poses significant challenges to the stability of reversibly assembled supramolecular photosensitizers that rely on non-covalent interactions, thereby limiting their practical therapeutic efficacy [28].

Compared to other macrocyclic host molecules, cucurbit[n]urils (Q[n]s) exhibit unique advantages due to their hydrophobic cavities and the electrostatic enrichment effect of the carbonyl oxygens at their portals, enabling the formation of stable host–guest complexes and maintaining structural integrity in complex pathological environments [29,30,31,32,33]. Notably, Q[n]s typically demonstrate high binding affinities toward cationic guests, with binding constants generally ranging from 10^5^ to 10^7^ M^−1^ [34,35]. In the present study, we selected partially methyl-substituted cucurbit[6]uril (HMeQ[6]) as the host molecule. Compared to cucurbiturils previously employed in the construction of porphyrin-based supramolecular photosensitizers [36,37,38], HMeQ[6] offers enhanced water solubility and a critical structural feature: the synergistic effect of a smaller portal size combined with a larger outer diameter (Table 1) allows it to maintain high host–guest binding affinity while effectively increasing steric hindrance between guest molecules, thereby inhibiting porphyrin aggregation [39,40]. Furthermore, we designed and synthesized a porphyrin derivative, DPPY, bearing two butyl cationic groups as the guest molecule. The selection of alkyl chain length is a critical factor. When the alkyl chain is excessively short (e.g., methyl or ethyl), HMeQ[6] may encapsulate the alkyl chains of different guest molecules at both termini, potentially leading to the formation of larger supramolecular polymers [41,42], which is detrimental to cellular uptake. Conversely, if the alkyl chain is excessively long, Q[6] tends to encapsulate only the distal end of the alkyl chain, thereby limiting its ability to effectively regulate the aggregation behavior of the porphyrin core. Consequently, butyl was chosen as the cationic substituent to facilitate effective host-guest encapsulation while inhibiting porphyrin aggregation, ultimately optimizing the photophysical properties and biocompatibility of the system. By exploiting the host–guest interactions between DPPY and HMeQ[6], we successfully constructed a novel supramolecular photosensitizer. The host-guest interactions between HMeQ[6] and DPPY were systematically investigated using multiple techniques, including ^1^H NMR titration, UV-Vis absorption spectroscopy, and fluorescence spectroscopy. Additionally, the photophysical properties of the supramolecular complex HMeQ[6]@DPPY, as well as its capacity to generate ^1^O_2_, were thoroughly examined. In this system, HMeQ[6] encapsulates the cationic side chains of DPPY, effectively suppressing DPPY aggregation, which reduces non-radiative transition processes and significantly enhances the generation efficiency of ^1^O_2_, thereby demonstrating excellent photophysical properties and activation characteristics. Subsequently, mouse breast cancer 4T1 cells were employed as an in vitro model. The cellular uptake of the supramolecular photosensitizer was monitored in real time using laser confocal microscopy. Intracellular ROS generation was quantified utilizing the DCFH-DA fluorescent probe. Additionally, cell viability was evaluated via the CCK-8 assay, and apoptosis was assessed through flow cytometry. Concurrently, key oxidative stress markers—including lipid peroxide (LPO), reduced glutathione (GSH) levels, and superoxide dismutase (SOD) activity—were measured using specific assay kits. Collectively, these analyses provided a comprehensive evaluation of the photodynamic therapy efficacy and in vitro cytotoxicity of the supramolecular photosensitizer. At the cellular level, the complex exhibits rapid internalization and favorable intracellular distribution, efficiently producing ROS upon light irradiation, which induces concentration-dependent oxidative stress and apoptosis. Based on these mechanisms, the constructed supramolecular polymer displays enhanced phototoxicity and cytotoxicity against 4T1 cells, substantially improving the efficacy of PDT. Compared to the conventional approach of enhancing porphyrin water solubility and PDT efficiency by incorporating large, sterically hindered hydrophilic substituents onto the porphyrin backbone via organic synthesis, the host-guest supramolecular engineering strategy is simpler and more direct, as it does not necessitate complex multi-step organic synthesis. This study thus provides a novel strategy for developing efficient supramolecular photosensitizers and holds significant potential for advancing PDT applications in cancer treatment.

## 2. Results and Discussion

**Figure 1:**
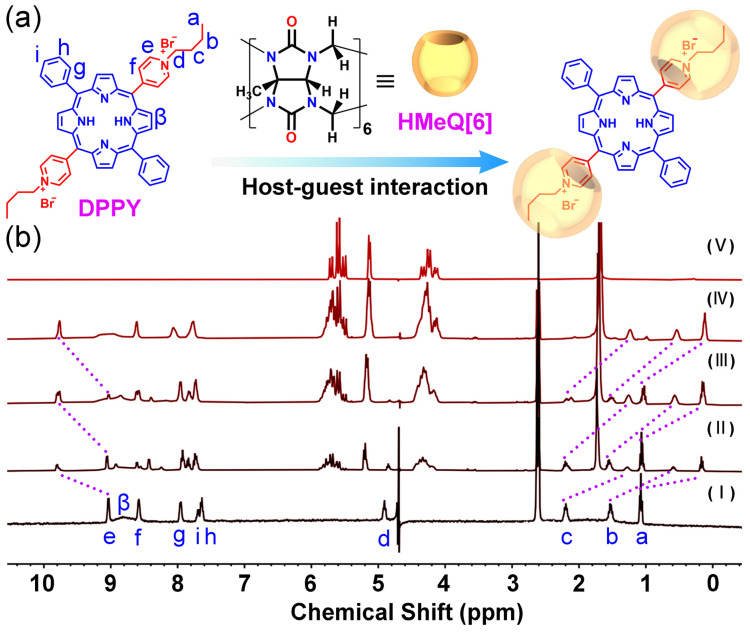
(**a**) Proposed interaction between DPPY and HMeQ[6]. (**b**) ^1^H NMR titration spectra of DTPP (pH = 6.52, 1 mM) with varying molar equivalents of HMeQ[6]: (I) 0.00, (II) 0.2, (III) 1.0, (IV) 2.0, and (V) HMeQ[6] alone, recorded in a mixed solvent system comprising 10% DMSO and 90% D_2_O.

Initially, the DPPY was synthesized via the reaction of 5,15-diphenyl-10,20-di(pyridin-4-yl)porphyrin with 1-bromobutane (Scheme S1). The molecular structure of DPPY was confirmed by ^1^H NMR spectroscopy and electrospray ionization mass spectrometry (ESI-MS) (Figures S1 and S2). Subsequently, the host–guest interaction between DPPY and HMeQ[6] was examined in aqueous solution using ^1^H NMR spectroscopy [38]. As illustrated in Figure 1, the addition of HMeQ[6] induced changes in the microenvironment of DPPY, resulting in notable chemical shift variations in its proton signals. Proton assignments on DPPY were corroborated by correlation spectroscopy (COSY) following titration (Figure S3). Specifically, under the shielding influence of the HMeQ[6] cavity, the Ha proton on the cationic arm of DPPY exhibited an upfield shift from δ = 1.01 to 0.12 ppm; Hb shifted from δ = 1.52 to 0.54 ppm; and Hc shifted from δ = 2.19 to 1.24 ppm. Concurrently, the He proton on the pyridinium ring experienced a downfield shift from δ = 9.05 to 9.76 ppm, attributable to interaction with the carbonyl oxygen at the portal of HMeQ[6]. These observations indicate that the cationic arms and a portion of the pyridinium ring of DPPY are encapsulated within the HMeQ[6] cavity, whereas the central porphyrin ring remains external to the cavity. Upon reaching a molar equivalent of 2.0 for HMeQ[6], further addition did not elicit additional chemical shift changes in DPPY protons, suggesting a host–guest stoichiometric ratio of 2:1 (HMeQ[6]:DPPY). This stoichiometry was further confirmed by a Job’s plot derived from DPPY fluorescence data, which exhibited an inflection point at a molar fraction of 0.66 (Figure S4). According to the Job’s plot, the stoichiometric ratio of host to guest is given by (1 − X_max_):X_max_, where X_max_ represents the molar fraction at the maximum point. In this case, with X_max_ equal to 0.66, the stoichiometry is calculated as (1 − 0.66):0.66 = 0.34:0.66. This ratio simplifies to approximately 1:2, indicating that two HMeQ[6] host molecules associate with one DPPY guest molecule. Moreover, analysis of the UV–visible absorption intensity of DPPY at 418 nm as a function of initial HMeQ[6] concentration, fitted according to the Benesi–Hildebrand equation (Figure S5), yielded the linear relationship y = 0.70 − 3.32 × 10^−6^x with a correlation coefficient (R^2^) of 0.991. From this, the binding constant (K_a_) for the DPPY–HMeQ[6] complex in aqueous solution was determined to be 2.11 × 10^5^ M^−1^. Collectively, these findings demonstrate the formation of a DPPY@HMeQ[6] complex mediated by specific interactions between HMeQ[6] and DPPY.

**Figure 2:**
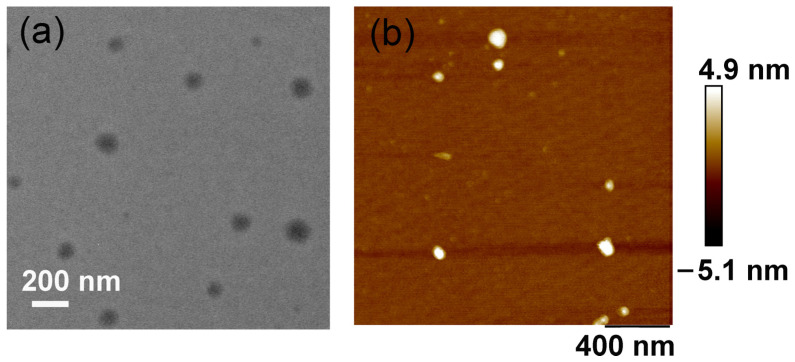
(**a**) TEM and (**b**) AFM images of DPPY@HMeQ[6] ([DTPP@Q[7]] = 20 μM).

Transmission Electron Microscopy (TEM) and Atomic Force Microscopy (AFM) analyses provided compelling evidence for the formation of supramolecular assemblies. Following the formation of the host-guest complex, TEM images revealed nanosheets composed of smaller subassemblies, with dimensions of approximately 100 nm (Figure 2a). AFM measurements corroborated these observations, indicating that the host-guest complex formed nanosheets with a thickness of roughly 10 nm, consistent with layered structures assembled from the DPPY@HMeQ[6] complex (Figure 2b). Furthermore, Dynamic Light Scattering (DLS) and zeta potential analyses furnished additional supporting data. The hydrodynamic diameter of the supramolecular complex increased markedly from 15.9 nm to 79.5 nm (Figure S6). Concurrently, the zeta potential of the complex increased to 15.2 ± 2.1 mV, compared to 13.0 ± 2.8 mV for free DPPY (Figure S7). The enhanced positive surface charge of the complex is likely to facilitate stronger interactions with negatively charged proteins on the cancer cell membrane, thereby promoting its accumulation within cancer cells. Collectively, these findings confirm that, subsequent to host-guest complex formation, DPPY@HMeQ[6] further assembles into larger supramolecular structures. This phenomenon can be attributed to the outer wall properties of HMeQ[6], which is not only positively charged but also capable of interacting with negatively charged entities to form larger self-assembled aggregates [43,44].

**Figure 3:**
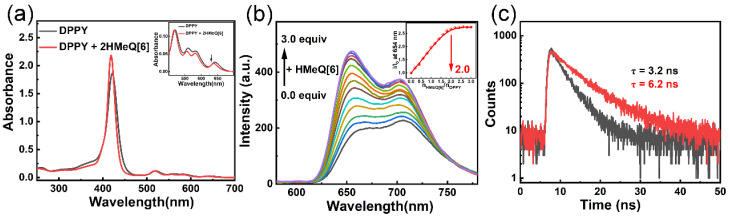
(**a**) UV-Vis absorption spectra of DPPY and DPPY@HMeQ[6] at pH 6.52 and a concentration of 20 μM. Inset: Absorbance in the range of 500–700 nm. (**b**) Fluorescence spectra of DPPY (pH 6.52, 20 μM) with increasing concentrations of HMeQ[6] from 0 to 3.0 equivalents. Inset: Variation in fluorescence intensity at 654 nm as a function of the molar ratio NHMeQ[6]/NDPPY. (**c**) Fluorescence lifetimes of DPPY and DPPY@HMeQ[6].

We investigated whether the host–guest complexation between DPPY and HMeQ[6] induces alterations in the photophysical properties of DPPY. As illustrated in Figure 3a, in aqueous solution, the presence of HMeQ[6] caused a slight blue shift in the Q-band absorption spectrum of DPPY, accompanied by a decrease in absorbance. This phenomenon is primarily attributed to the insertion of the cationic arm of DPPY into the nonpolar cavity of HMeQ[6], whereby the carbonyl oxygen at the portal attenuates the electron-withdrawing effect exerted by the pyridinium ion. Notably, the S-band absorption at 425 nm, corresponding to the strong absorption of the porphyrin core, exhibited a marked enhancement. Given that the molar extinction coefficient of chromophores depends on the spatial separation between adjacent chromophores, the observed increase in porphyrin absorption suggests an increased distance between porphyrin units. In other words, encapsulation of the cationic arm of DPPY by HMeQ[6] disrupts the π–π interactions among porphyrin molecules, thereby inhibiting aggregation of the porphyrin cores. As shown in Figure 3b, due to ACQ effects in aqueous media, the fluorescence emission spectrum of DPPY, upon excitation at 520 nm, displays a broad and subdued peak between 610 and 780 nm, characteristic of self-quenching [45,46]. However, upon addition of HMeQ[6], the fluorescence emission spectrum of DPPY resolves into two distinct peaks with enhanced intensities at 654 nm and 719 nm. Specifically, the fluorescence intensity at 654 nm increased significantly from 172.5 a.u. to 469.8 a.u., representing an enhancement of approximately threefold. Concurrently, complexation with HMeQ[6] resulted in a moderate increase in the fluorescence quantum yield of DPPY from 2.8% to 3.8% (Figure S8), and the excited-state lifetime measured at 654 nm extended from 3.2 ns to 6.2 ns (Figure 3c). Beyond suppressing porphyrin aggregation, HMeQ[6] encapsulation of the cationic arm also disrupts intramolecular charge transfer between the pyridyl group and the porphyrin core, leading to modifications in the excited-state electron distribution that may account for the observed fluorescence changes upon host–guest complexation. Furthermore, the rigid cavity of HMeQ[6] provides a more hydrophobic microenvironment that inhibits nonradiative decay pathways of DPPY.

**Figure 4:**
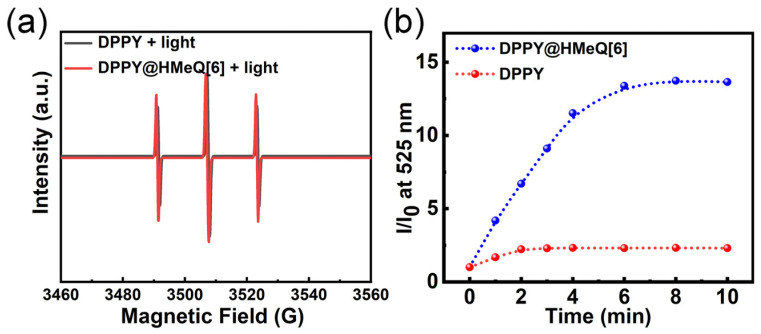
(**a**) EPR spectra recorded after 10 min of irradiation of DPPY and DPPY@HMeQ[6]. (**b**) Changes in the fluorescence intensity of SOSG in the presence of DPPY and DPPY@HMeQ[6] (pH = 6.52, 20 μM) following irradiation with a 520 nm laser beam (500 mW/cm^2^) over various time intervals.

The ^1^O_2_ generation capability of the DPPY@HMeQ[6] complex, functioning as a photosensitizer, was systematically evaluated by assessing its ^1^O_2_ production efficiency. Electron paramagnetic resonance (EPR) spectroscopy was employed to monitor ^1^O_2_ generation. The spin-trapping agent 2,2,6,6-tetramethylpiperidine (TEMP) was utilized as the EPR probe due to its ability to react with ^1^O_2_, forming the paramagnetic nitroxide radical TEMPO [47,48]. Under dark conditions, the EPR spectra of DPPY@HMeQ[6] and free DPPY were comparable (Figure S9). However, following 10 min of light irradiation, the EPR signal intensity of the DPPY@HMeQ[6] complex was markedly greater than that of DPPY alone (Figure 4a). Subsequently, the ^1^O_2_ generation efficiency was further quantified using the commercial probe Singlet Oxygen Sensor Green (SOSG), which is selectively oxidized by ^1^O_2_ and emits green fluorescence at 525 nm. As depicted in Figure 4b, upon irradiation with a 520 nm laser (500 mW/cm^2^), changes in SOSG fluorescence (Figures S10 and S11) were recorded, and the fluorescence intensity versus irradiation time curve revealed distinct kinetic differences. Comparative analysis of the slopes indicated that the ^1^O_2_ generation rate of the DPPY@HMeQ[6] complex was substantially higher than that of free DPPY. Moreover, after 10 min of laser exposure, the amount of ^1^O_2_ produced by DPPY@HMeQ[6] was approximately sixfold greater than that generated by free DPPY. The host–guest interaction between DPPY and HMeQ[6] effectively inhibited aggregation of the DPPY photosensitizer in aqueous solution, thereby significantly enhancing its ^1^O_2_ generation efficiency. Consequently, DPPY@HMeQ[6] demonstrates the properties of a supramolecular photosensitizer.

**Figure 5:**
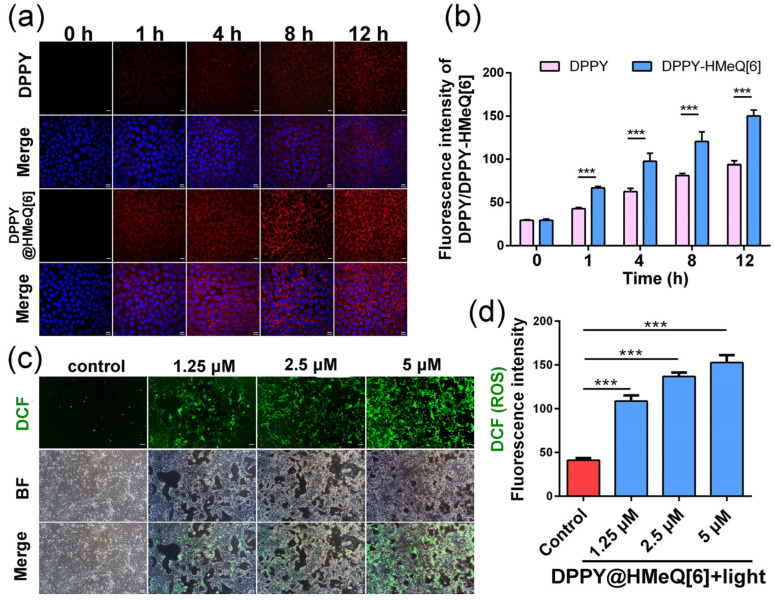
(**a**) Cellular uptake of DPPY and DPPY@HMeQ[6] in 4T1 cells. Scale bar: 10 μm. (**b**) Corresponding quantification of fluorescence intensity (n = 10). (**c**) Intracellular ROS levels in 4T1 cells following treatment with varying concentrations of DPPY and DPPY@HMeQ[6] under 520 nm laser irradiation for 3 min. Scale bar: 20 μm. (**d**) Corresponding quantification of ROS levels (n = 10). Data are presented as mean ± standard deviation (SD). Statistical significance was determined at *p* < 0.05. (*** *p* < 0.001).

Based on the activatable properties of the DPPY@HMeQ[6] complex, it is hypothesized that this system may be suitable for applications in bioimaging and PDT. To investigate this potential, we first assessed the cellular uptake of the supramolecular photosensitizer by 4T1 cells. 4T1 cells are derived from a mouse triple-negative breast tumor and exhibit pronounced invasive and metastatic properties, rendering them an effective model for replicating the pathological progression of human triple-negative breast cancer [19,49]. During this evaluation, the nuclear dye DAPI was employed as an internal reference, and fluorescence signals were detected using two channels via confocal laser scanning microscopy (CLSM). As illustrated in Figure 5a, the blue fluorescence channel distinctly delineates the cell nuclei, whereas the red fluorescence signal originates from the emission of DPPY, indicating that both free DPPY and the DPPY@HMeQ[6] complex predominantly localize within the cytoplasm. Notably, red fluorescence from free DPPY was observed only after 2 h of incubation, whereas the DPPY@HMeQ[6] complex exhibited pronounced red fluorescence after merely 1 h of incubation. This observation suggests that complexation with HMeQ[6] significantly enhances the fluorescence intensity of DPPY, corroborating previous fluorescence enhancement studies. With extended incubation, the red fluorescence intensity progressively increased, further confirming the effective uptake of the DPPY@HMeQ[6] complex by 4T1 cells (Figure 5b). Subsequently, intracellular ROS generation efficiency under light irradiation was evaluated using the ROS probe 2,7-dichlorofluorescein diacetate (DCFH-DA). This probe is oxidized by ROS to emit green fluorescence, the intensity of which correlates positively with intracellular ROS levels. After 3 min of irradiation with a 520 nm laser, inverted fluorescence microscopy (IFM) images (Figure 5c) demonstrated that, relative to the control group, cells treated with varying concentrations of DPPY@HMeQ[6] under light exposure exhibited green fluorescence, with fluorescence intensity increasing in a concentration-dependent manner (Figure 5d). Flow cytometry analysis further revealed that the ROS-associated fluorescence peak increased with higher concentrations of DPPY@HMeQ[6] (Figure S12). Collectively, these results indicate that the supramolecular photosensitizer exhibits efficient ROS generation under light irradiation, consistent with in vitro ROS detection assays.

**Figure 6:**
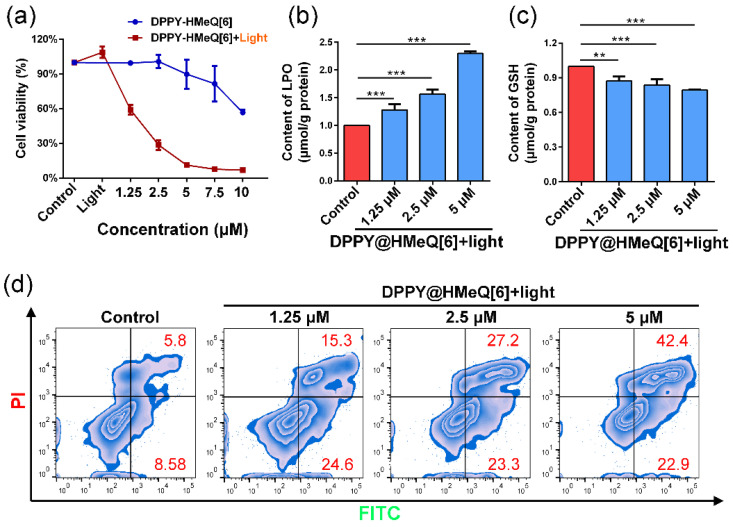
(**a**) Cell viability of 4T1 cells following treatment with varying concentrations of DPPY@HMeQ[6] under 520 nm laser irradiation for 3 min (n = 3). (**b**) GSH and (**c**) LPO levels in 4T1 cells after drug treatment (n = 3). (**d**) Flow cytometric analysis of apoptosis in 4T1 cells post-treatment. Data are presented as mean ± SD. Statistical significance was determined at *p* < 0.05. (** *p* < 0.01, *** *p* < 0.001).

Building upon the aforementioned research findings, we further investigated the therapeutic efficacy of DPPY@HMeQ[6] as a PDT photosensitizer in live cancer cells. Initially, the effect of DPPY@HMeQ[6] on the viability of 4T1 cells was evaluated using the Cell Counting Kit-8 (CCK-8) assay. As depicted in Figure 6a, under dark conditions without laser irradiation, DPPY@HMeQ[6] demonstrated minimal dark toxicity over a concentration range of 0 to 5 μM, with cell viability consistently exceeding 90%. Conversely, upon exposure to 520 nm laser irradiation (500 mW/cm^2^, 3 min), 4T1 cell viability decreased significantly in a dose-dependent manner with increasing concentrations of DPPY@HMeQ[6], yielding an IC50 value of 1.8 μM (Figure S13), indicative of substantial phototoxicity of the complex. Furthermore, an elevation in LPO levels was observed (Figure 6b), accompanied by depletion of GSH, a critical antioxidant molecule, and a compensatory increase in SOD activity (Figure S14). In contrast, HMeQ[6] alone demonstrated no significant cytotoxicity within the concentration range of 0 to 10 μM (Figure S15). Collectively, these results suggest that the supramolecular photosensitizer is activated upon light exposure within the cells, leading to increased ROS generation, which subsequently induces oxidative stress and apoptosis. To confirm this mechanism, apoptosis was assessed via FITC-Annexin V/PI double staining coupled with flow cytometry. The data (Figure 6d and Figure S16) revealed that the apoptosis rate of 4T1 cells increased from 39.9% to 65.3% with rising concentrations of DPPY@HMeQ[6], correlating with intracellular ROS level changes. In summary, HMeQ[6], through host-guest interactions with DPPY, effectively inhibits DPPY molecular aggregation, thereby enhancing its ^1^O_2_ generation under 520 nm laser irradiation and significantly promoting oxidative stress and apoptosis in 4T1 cells.

## 3. Materials and Methods

### 3.1. Materials

All materials listed below were of analytical purity grade and were utilized without further purification. 10,20-diphenyl-5,15-dipyridin-4-yl-21,22-dihydroporphyrin and 1-Bromobutane was obtained from Aladdin (Shanghai, China). HMeQ[6] was provided by the Key Laboratory of Macrocyclic and Supramolecular Chemistry of Guizhou Province (Guiyang, China). Dulbecco’s Modified Eagle Medium (DMEM) and penicillin-streptomycin were purchased from Gibco (Waltham, MA, USA). Fetal bovine serum (FBS) was acquired from Umedium (Hefei, China). Phosphate-buffered saline (PBS) and phenylmethanesulfonyl fluoride (PMSF) were obtained from Solarbio (Beijing, China). DAPI, CCK-8, Calcein-AM/PI live/dead cell staining kit, SOSG, DCFH-DA, and RIPA protein lysate were purchased from Beyotime (Shanghai, China). Glutathione (GSH), malondialdehyde (MDA), and lipid peroxidation (LPO) assay kits were obtained from Jiancheng (Nanjing, China). The apoptosis assay kit was acquired from KeyGEN (Nanjing, China).

### 3.2. Characterization

A JEOL JNM-ECZ400S nuclear magnetic resonance spectrometer (JEOL, Tokyo, Japan, 400 MHz) was employed to acquire ^1^H NMR spectra. Ultraviolet-visible (UV-Vis) spectroscopy data were obtained using a SHIMADZU UV-2600i spectrophotometer (Shimadzu, Kyoto, Japan). Transmission electron microscopy (TEM) images were captured with a FET Talos F200C field emission transmission electron microscope (Thermo Fisher Scientific, Waltham, MA, USA). Dynamic light scattering (DLS) measurements were conducted using a NanoBrook 90Plus instrument (Brookhaven, New York, NY, USA). Fluorescence quantum yields were determined with an FLS1000 Photoluminescence Spectrometer (Edinburgh, UK). Laser confocal microscopy was performed using an Olympus Spin 10 system (Olympus, Tokyo, Japan). Inverted fluorescence microscopy was carried out with Nikon TI-SR (Nikon, Tokyo, Japan) and Shunyu ICX41 (Shunyu, Yuyao, China) instruments. Flow cytometry analysis was conducted using a BD FACSCelesta (BD, Franklin Lakes, NJ, USA). A microplate reader (Allsheng, Hangzhou, China) was utilized for relevant experimental measurements.

### 3.3. Synthesis of DPPY

10,20-Diphenyl-5,15-dipyridin-4-yl-21,22-dihydroporphyrin (154 mg, 0.25 mmol) and 1-bromobutane (680 mg, 5 mmol) were dissolved in 10 mL of dimethylformamide (DMF). The resulting solution was stirred at 130 °C for 12 h under a nitrogen atmosphere. After this period, the solution was allowed to cool and was subsequently filtered. The solid precipitate was thoroughly washed with trichloromethane and diethyl ether, then dried under vacuum. The overall yield of the reaction was 70%. The ^1^H NMR spectrum (400 MHz, DMSO) showed signals at δ (TMS, ppm): 9.55 (d, 2H), 9.10 (d, 2H), 9.04 (d, 2H), 8.96 (d, 2H), 8.24 (d, 2H), 8.00–7.80 (m, 3H), 4.95 (t, 2H), 2.26 (t, 2H), 1.63 (t, 2H), and 1.12 (t, 3H). Electrospray ionization mass spectrometry (ESI-MS) analysis yielded *m*/*z* [M-2Br]^2+ calculated as 365.1857, with an observed value of 368.1855.

### 3.4. ^1^H NMR Measurements

The ^1^H NMR spectra were acquired at 25 °C. Deuterium oxide (D2O) was utilized for field-frequency locking, and the chemical shifts were reported in parts per million (ppm) relative to the internal standard tetramethylsilane (TMS), which was assigned a value of 0 ppm for ^1^H. ^1^H NMR titrations were performed using solutions with a constant concentration of DPPY (1.00 mM, 500 μL) while varying the concentrations of HMeQ[6].

### 3.5. DPPY@HMeQ[6] Preparaton

Both ultraviolet and fluorescence spectroscopy data were employed to characterize the properties of two sets of solutions. The first set was prepared using the molar ratio method, wherein varying amounts of HMeQ[6] (0, 0.2, 0.4, …, 3.0 equivalents) were added to a fixed concentration of DPPY (20 μM). The second set was prepared according to the Job method and comprised ten pairs of control solutions (20 μM) corresponding to different molar ratios of NHMeQ[6] to the combined NHMeQ[6] and DPPY, specifically at ratios of 0.1, 0.2, 0.3, 0.4, 0.5, 0.6, 0.7, 0.8, 0.9, and 1.0. Fluorescence spectrometry measurements were conducted with an excitation wavelength of 520 nm, a slit width of 10 nm, and a photomultiplier tube voltage of 700 V.

### 3.6. Singlet Oxygen Generation

The concentration of singlet oxygen generated in solution was evaluated. A 5 mM solution of Singlet Oxygen Sensor Green (SOSG, Biyuntian, Shanghai, China) was prepared by dissolving the compound in methanol; this solution was freshly prepared at the start of each experiment. DPPY and DPPY@HMeQ[6] complexes, with a DPPY concentration of 20.0 µM, were mixed with SOSG at a concentration of 5 µM in a 5 mM sodium phosphate buffer adjusted to pH 6.52. The samples were irradiated using a laser at 520 nm with an intensity of 500 mW/cm^2^. Fluorescence measurements of SOSG were recorded at one-minute intervals by excitation at 495 nm and emission collection between 505 and 650 nm.

### 3.7. Cell Culture and Cell Experiment

4T1 cell line was procured from GAINING (Shanghai, China; Catalog No. CM-M029). Cells were cultured in high-glucose Dulbecco’s Modified Eagle Medium (DMEM) supplemented with 10% fetal bovine serum (FBS) and 1% penicillin-streptomycin. Cultures were maintained in a humidified incubator at 37 °C with 5% CO_2_.

#### 3.7.1. Cellular Uptake

4T1 cells were seeded into laser confocal cell culture dishes. After a 24-h incubation period, the culture medium was replaced with DMEM supplemented with DPPY and DPPY@HMeQ[6] at a concentration of 20 µM. The cells were then incubated with these compounds for varying durations. Finally, the cells were stained with DAPI. Fluorescence imaging was performed using a confocal laser scanning microscope (CLSM) with an excitation wavelength of 561 nm.

#### 3.7.2. Cytotoxicity Test

Cell viability was assessed using the CCK-8 assay. Cells were seeded in a 96-well culture plate at a density of 8 × 10^3^ cells per well in 100 μL of growth medium and incubated for a specified period. Following incubation, the medium was aspirated, and the cells were treated with varying concentrations (0, 1.25, 2.5, 5, 7.5, and 10 μM) of T DPPY and DPPY@HMeQ[6], diluted in fresh medium. For the light treatment groups, cells were exposed to a 520 nm laser at an intensity of 500 mW/cm^2^ for 3 min after a 4-h incubation with the compounds. Subsequently, 10 μL of CCK-8 solution was added to each well, and the plates were incubated for 1 h. Absorbance was then measured at 450 nm using a microplate reader to determine cell viability.

#### 3.7.3. ROS Assay

Cells were seeded into 24-well plates and incubated overnight under standard cell culture conditions. Subsequently, the cells were treated with varying concentrations of DPPY@HMeQ[6] (1.25, 2.5, and 5 μM) and incubated for 4 h. Following this incubation, the cells were exposed to irradiation using a 520 nm laser at an intensity of 500 mW/cm^2^ for 3 min. After an additional 16-h incubation period, each well was washed three times with phosphate-buffered saline (PBS), and 1 mL of 15 μM DCFH-DA diluted in DMEM was added. The plates were then returned to the incubator for 30 min. Subsequently, the cells were washed three times with PBS, 1 mL of DMEM was added, and the cells were examined using an inverted fluorescence microscope.

#### 3.7.4. Detection of GSH, Reduced SOD and LPO

Cells were cultured overnight in 100 mm dishes within a cell incubator. Subsequently, varying concentrations of DPPY@HMeQ[6] (1.25, 2.5, and 5 μM) were administered to the cells. Following a 4-h incubation period, the samples designated for light exposure were irradiated with a 520 nm laser at an intensity of 500 mW/cm^2^ for 10 min. After an additional 24-h incubation, the cells were collected by centrifugation at 2000 rpm for 5 min and washed three times with phosphate-buffered saline (PBS). Thereafter, 300 μL of RIPA protein lysis buffer containing 1% PMSF was added to each tube. Cell lysis was performed using an ultrasonic homogenizer set to 120 W, applying cycles of 10 s sonication followed by 10 s pause, repeated three times. The lysates were then incubated on ice for 30 min to ensure complete lysis. Subsequently, the samples were centrifuged at 12,000 rpm for 25 min, and the supernatants were collected for analysis in accordance with the manufacturer’s protocol. Absorbance measurements were obtained using a microplate reader.

#### 3.7.5. Annexin V-FITC/PI Apoptosis Assay

Cells were cultured overnight in 60 mm dishes within a cell incubator. Subsequently, varying concentrations of DPPY@HMeQ[6] (1.25, 2.5, and 5 μM) were administered to the cells. After a 4-h incubation period, the samples designated for light treatment were irradiated with a 520 nm laser at an intensity of 500 mW/cm^2^ for 5 min. Following an additional 24-h incubation, the cells were collected by centrifugation at 2000 rpm for 5 min and washed three times with phosphate-buffered saline (PBS). Each sample tube was then supplemented with 500 μL of staining buffer, 5 μL of fluorescein isothiocyanate (FITC), and 5 μL of propidium iodide (PI), except for the single-stained and blank control tubes. These samples were incubated at room temperature for 15 min. Finally, fluorescence intensity was assessed using flow cytometry.

#### 3.7.6. Statistical Analysis

The experimental results are presented as mean ± standard deviation (SD). All experiments were performed in triplicate, consisting of three independent trials. Statistical significance between two groups was assessed using the Student’s *t*-test, while differences among multiple groups were evaluated by one-way analysis of variance (ANOVA). Data analysis and figure preparation were conducted using GraphPad Prism (version 8.0.2) and Origin 2021 software. A *p*-value less than 0.05 was considered indicative of statistical significance.

## 4. Conclusions

This study represents the first application of HMeQ[6], a compound characterized by favorable water solubility, to construct a complex with the porphyrin derivative DPPY via host-guest interactions, resulting in the novel supramolecular photosensitizer DPPY@HMeQ[6]. Comprehensive characterization techniques, including ^1^H NMR, fluorescence spectroscopy, UV–vis spectroscopy, TEM, and DLS, confirmed that the host and guest molecules associate in a 2:1 molar ratio to form the complex, exhibiting a K_a_ of 2.11 × 10^5^ M^−1^. Furthermore, the complex self-assembles into nanosheet structures. Notably, host-guest encapsulation substantially inhibits the aggregation of porphyrin moieties, thereby enhancing fluorescence emission intensity, excited-state lifetime, and ^1^O_2_ generation efficiency, indicative of superior photophysical activation. At the cellular level, the complex demonstrates rapid internalization and favorable intracellular distribution, efficiently generating ROS upon light irradiation. This process induces concentration-dependent oxidative stress and apoptosis, confirming the potential as a photosensitizer for PDT. Additionally, the complex exhibits minimal cytotoxicity under dark conditions, suggesting good biocompatibility. This work broadens the application of methyl-substituted cucurbiturils in functional supramolecular assemblies and validates the efficacy of host-guest encapsulation in modulating the performance of porphyrin-based photosensitizers. Compared to the previously reported TMeQ[6]-based supramolecular photosensitizer, DPPY@HMeQ[6] demonstrates increased phototoxicity, as evidenced by a lower IC_50_ value. This enhancement is primarily attributed to the larger cavity size and greater peripheral steric hindrance of HMeQ[6], which more effectively inhibit the aggregation of porphyrin molecules upon encapsulation of DPPY. Consequently, this inhibition improves the efficiency of ^1^O_2_ generation, thereby augmenting the photodynamic cytotoxicity against tumor cells. This system holds promising potential for bioimaging and PDT applications, offering novel insights for the development of advanced supramolecular photosensitizers.

## Figures and Tables

**Table 1:** Structural and Physical Parameters of Q[6], TMeQ[6], HMeQ[6] and Q[7].

	Q[6]	TMeQ[6]	HMeQ[6]	Q[7]
structure	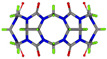	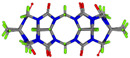	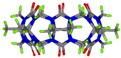	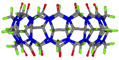
cavity diameter (Å)	5.8	6.2	5.9	7.3
outer diameter (Å)	14.4	14.9	15.1	16.0
solubility (mM)	0.018	0.1	0.5–1	20−30

## Data Availability

The original data is available from the corresponding author upon reasonable request.

## Supplementary Materials

The following supporting information can be downloaded at: https://www.mdpi.com/article/10.3390/molecules30234576/s1. Scheme S1: Synthesis of the guest DPPY; Figure S1: ^1^H NMR of DPPY; Figure S2: ESI-MS spectrum of DPPY; Figure S3: COSY spectrum of DPPY@HMeQ[6]; Figure S4: Job’s plot of DPPY and HMeQ[6]; Figure S5: Benesi−Hilderbrand plot of DPPY with HMeQ[6]; Figure S6: DLS of DPPY and DPPY@HMeQ[6]; Figure S7: Zeta potentials of DPPY and DPPY@HMeQ[6]; Figure S8: Fluorescence quantum yield of DPPY and DPPY@HMeQ[6]; Figure S9: EPR spectra of DPPY and DPPY@HMeQ[6]; Figure S10: FL intensity of SOSG in the presence of DPPY; Figure S11: FL intensity of SOSG in the presence of DPPY@HMeQ[6]; Figure S12: Flow cytometry images of ROS levels obtained through DCFH-DA staining; Figure S13: The IC50 of 4T1 cells treated with different doses of DPPY@HMeQ[6] and lighting; Figure S14: The content of SOD in 4T1 cells after drug intervention; Figure S15: Cell viability of 4T1 cells assessed after incubation with DPPY@HMeQ[6]; Figure S16: Corresponding statistical analysis of apoptosis.

## References

[1] Li X., Lovell J.F., Yoon J., Chen X. (2020). Clinical development and potential of photothermal and photodynamic therapies for cancer. Nat. Rev. Clin. Oncol..

[2] Pham T.C., Nguyen V.-N., Choi Y., Lee S., Yoon J. (2021). Recent Strategies to Develop Innovative Photosensitizers for Enhanced Photodynamic Therapy. Chem. Rev..

[3] van Straten D., Mashayekhi V., de Bruijn H.S., Oliveira S., Robinson D.J. (2017). Oncologic Photodynamic Therapy: Basic Principles, Current Clinical Status and Future Directions. Cancers.

[4] Yu L., Liu Z., Xu W., Jin K., Liu J., Zhu X., Zhang Y., Wu Y. (2024). Towards overcoming obstacles of type II photodynamic therapy: Endogenous production of light, photosensitizer, and oxygen. Acta Pharm. Sin. B.

[5] Sarbadhikary P., George B.P., Abrahamse H. (2021). Recent Advances in Photosensitizers as Multifunctional Theranostic Agents for Imaging-Guided Photodynamic Therapy of Cancer. Theranostics.

[6] Kwon S., Ko H., You D.G., Kataoka K., Park J.H. (2019). Nanomedicines for Reactive Oxygen Species Mediated Approach: An Emerging Paradigm for Cancer Treatment. Acc. Chem. Res..

[7] Maharjan P.S., Bhattarai H.K. (2022). Singlet Oxygen, Photodynamic Therapy, and Mechanisms of Cancer Cell Death. J. Oncol..

[8] Hu J.-J., Chen Y., Lou X., Xia F., Wu X., Yoon J. (2025). Recent strategies for developing membrane-targeting photodynamic therapy. Coord. Chem. Rev..

[9] Fang L., Chen Z., Dai J., Pan Y., Tu Y., Meng Q., Diao Y., Yang S., Guo W., Li L. (2025). Recent Advances in Strategies to Enhance Photodynamic and Photothermal Therapy Performance of Single-Component Organic Phototherapeutic Agents. Adv. Sci..

[10] Yang F., Xu M., Chen X., Luo Y. (2023). Spotlight on porphyrins: Classifications, mechanisms and medical applications. Biomed. Pharmacother..

[11] Park J.M., Hong K.-I., Lee H., Jang W.-D. (2021). Bioinspired Applications of Porphyrin Derivatives. Acc. Chem. Res..

[12] Zhao X., Liu J., Fan J., Chao H., Peng X. (2021). Recent progress in photosensitizers for overcoming the challenges of photodynamic therapy: From molecular design to application. Chem. Soc. Rev..

[13] Bodedla G.B., Zhu X., Wong W.-Y. (2023). An overview on AIEgen-decorated porphyrins: Current status and applications. Aggregate.

[14] Rui L.-L., Cao H.-L., Xue Y.-D., Liu L.-C., Xu L., Gao Y., Zhang W.-A. (2016). Functional organic nanoparticles for photodynamic therapy. Chin. Chem. Lett..

[15] Zhou G., Zhong W., Chen Y., Wang Y., Li T., Hua J., Zhou Y., Li M., Gu N., Zhao Y. (2023). Mixed-valence gold-porphyrin two-dimensional coordination networks for repurposing of chrysotherapy. Biomaterials.

[16] Shi L., Sun Z., Richy N., Blanchard-Desce M., Mongin O., Paul F., Paul-Roth C.O. (2024). Giant Star-shaped meso-substituted Fluorescent Porphyrins with Fluorenyl-containing Arms Designed for Two-photon Oxygen Photosensitization. Chem. Eur. J..

[17] Abid S., Hassine S.B., Sun Z., Richy N., Camerel F., Jamoussi B., Blanchard-Desce M., Mongin O., Paul F., Paul-Roth C. (2021). Impact of Changing the Core in Tetrapyrrolic Dendrimers Designed for Oxygen Sensitization: New Fluorescent Phthalocyanine-Based Dendrimers with High Two-Photon Absorption Cross-sections. Macromolecules.

[18] Wei K., Wu Y., Zheng X., Ouyang L., Ma G., Ji C., Yin M. (2024). A Light-Triggered J-Aggregation-Regulated Therapy Conversion: From Photodynamic/Photothermal Therapy to Long-Lasting Chemodynamic Therapy for Effective Tumor Ablation. Angew. Chem. Int. Ed..

[19] Zhen W., Kang D.W., Fan Y., Wang Z., Germanas T., Nash G.T., Shen Q., Leech R., Li J., Engel G.S. (2024). Simultaneous Protonation and Metalation of a Porphyrin Covalent Organic Framework Enhance Photodynamic Therapy. J. Am. Chem. Soc..

[20] Rajora M.A., Lou J.W.H., Zheng G. (2017). Advancing porphyrin’s biomedical utility via supramolecular chemistry. Chem. Soc. Rev..

[21] Liu Y.-Y., Yu X.-Y., Pan Y.-C., Yin H., Chao S., Li Y., Ma H., Zuo M., Teng K.-X., Hou J.-L. (2024). Supramolecular systems for bioapplications: Recent research progress in China. Sci. China Chem..

[22] Zheng B.-D., Ye J., Zhang X.-Q., Zhang N., Xiao M.-T. (2021). Recent advances in supramolecular activatable phthalocyanine-based photosensitizers for anti-cancer therapy. Coord. Chem. Rev..

[23] Tian J., Huang B., Nawaz M.H., Zhang W. (2020). Recent advances of multi-dimensional porphyrin-based functional materials in photodynamic therapy. Coord. Chem. Rev..

[24] Song Y.-H., Gu Y.-J., Lei Z., Li N.-K., Zhang Y.-M., Yu Q., Liu Y. (2025). Fluorinated Cyclodextrin Supramolecular Nanoassembly Enables Oxygen-Enriched and Targeted Photodynamic Therapy. Nano Lett..

[25] Huang B., Wang P., Ouyang Y., Pang R., Liu S., Hong C., Ma S., Gao Y., Tian J., Zhang W. (2020). Pillar[5]arene-Based Switched Supramolecular Photosensitizer for Self-Amplified and pH-Activated Photodynamic Therapy. ACS Appl. Mater. Interfaces.

[26] Xiao B., Wang Q., Zhang S., Li X.-Y., Long S.-Q., Xiao Y., Xiao S., Ni X.-L. (2019). Cucurbit[7]uril-anchored polymer vesicles enhance photosensitization in the nucleus. J. Mater. Chem. B.

[27] Sun C., Zhang H., Yue L., Li S., Cheng Q., Wang R. (2019). Facile Preparation of Cucurbit[6]uril-Based Polymer Nanocapsules for Targeted Photodynamic Therapy. ACS Appl. Mater. Interfaces.

[28] Dai Y., Xu C., Sun X., Chen X. (2017). Nanoparticle design strategies for enhanced anticancer therapy by exploiting the tumour microenvironment. Chem. Soc. Rev..

[29] Li Q., Yu Z., Redshaw C., Xiao X., Tao Z. (2024). Double-cavity cucurbiturils: Synthesis, structures, properties, and applications. Chem. Soc. Rev..

[30] Hu J.-H., Huang Y., Redshaw C., Tao Z., Xiao X. (2023). Cucurbit[n]uril-based supramolecular hydrogels: Synthesis, properties and applications. Coord. Chem. Rev..

[31] Yan M., Wu S., Wang Y., Liang M., Wang M., Hu W., Yu G., Mao Z., Huang F., Zhou J. (2024). Recent Progress of Supramolecular Chemotherapy Based on Host–Guest Interactions. Adv. Mater..

[32] Muheyati M., Wu G., Li Y., Pan Z., Chen Y. (2024). Supramolecular nanotherapeutics based on cucurbiturils. J. Nanobiotechnol..

[33] Yang K., Zhang Z., Du J., Li W., Pei Z. (2020). Host–guest interaction based supramolecular photodynamic therapy systems: A promising candidate in the battle against cancer. Chem. Commun..

[34] Macartney D.H. (2018). Cucurbit[n]uril Host-Guest Complexes of Acids, Photoacids, and Super Photoacids. Isr. J. Chem..

[35] Kaifer A.E. (2014). Toward Reversible Control of Cucurbit[n]uril Complexes. Acc. Chem. Res..

[36] Xiao B., Liao Y., Zhang J., Chen K., Feng G., Feng J., Zhang C. (2024). Tetramethyl Cucurbit[6]uril±Porphyrin Supramolecular Polymer Enhances Photosensitization. Int. J. Mol. Sci..

[37] Lee J.-S., Song I.-H., Shinde P.B., Nimse S.B. (2020). Macrocycles and Supramolecules as Antioxidants: Excellent Scaffolds for Development of Potential Therapeutic Agents. Antioxidants.

[38] Hu H., Wang H., Yang Y., Xu J.-F., Zhang X. (2022). A Bacteria-Responsive Porphyrin for Adaptable Photodynamic/Photothermal Therapy. Angew. Chem. Int. Ed..

[39] Liu Q., Tang Q., Xi Y.-Y., Huang Y., Xiao X., Tao Z., Xue S.-F., Zhu Q.-J., Zhang J.-X., Wei G. (2015). Host–guest interactions of thiabendazole with normal and modified cucurbituril: 1H NMR, phase solubility and antifungal activity studies. Supramol. Chem..

[40] Meng L.-J., Tian X., Huang S., Lin R.-L., Liu X.-H., Zhu Q.-J., Tao Z., Liu J.-X. (2018). Solvent- and Heat-Dependent Binding Behaviors of HMeQ[6] with Alkyldiammonium Ions. ChemistrySelect.

[41] Zhang W., Luo Y., Zhao J., Lin W.-H., Ni X.-L., Tao Z., Xiao X., Xiao C.-D. (2022). tQ[14]-based AIE supramolecular network polymers as potential bioimaging agents for the detection of Fe^3+^ in live HeLa cells. Sens. Actuators B-Chem..

[42] Li F.-F., Huo M., Kong J., Liu Y. (2024). Cucurbituril-Confined Tetracation Supramolecular 2D Organic Framework for Dual-Emission TS-FRET. Adv. Opt. Mater..

[43] Liu C., Gao R., Zhang Y., Zhu Q., Tao Z. (2021). Cucurbit[6]uril-based supramolecular frameworks assembled via the outer surface interaction of cucurbit[n]urils. Chin. Chem. Lett..

[44] Huang Y., Gao R.-H., Liu M., Chen L.-X., Ni X.-L., Xiao X., Cong H., Zhu Q.-J., Chen K., Tao Z. (2021). Cucurbit[n]uril-Based Supramolecular Frameworks Assembled through Outer-Surface Interactions. Angew. Chem. Int. Ed..

[45] Wang H., Yang Y., Yuan B., Ni X.-L., Xu J.-F., Zhang X. (2021). Cucurbit[10]uril-Encapsulated Cationic Porphyrins with Enhanced Fluorescence Emission and Photostability for Cell Imaging. ACS Appl. Mater. Interfaces.

[46] Liu Y., Huang Z., Liu K., Kelgtermans H., Dehaen W., Wang Z., Zhang X. (2014). Porphyrin-containing hyperbranched supramolecular polymers: Enhancing ^1^O_2_-generation efficiency by supramolecular polymerization. Polym. Chem..

[47] Li Q., Zhang P., Wang P., Yan C., Wang K., Yang W., Dang D., Cao L. (2025). A combination of covalent and noncovalent restricted-intramolecular-rotation strategy for supramolecular AIE-type photosensitizer toward photodynamic therapy. Aggregate.

[48] Liu Y., Zhang J., Zhou X., Wang Y., Lei S., Feng G., Wang D., Huang P., Lin J. (2024). Dissecting Exciton Dynamics in pH-Activatable Long-Wavelength Photosensitizers for Traceable Photodynamic Therapy. Angew. Chem. Int. Ed..

[49] Zhou Q., Wang K., Yang D., Huang Y., Yu M., Xie D., Wang J., Chen S., Gong J., Yang M. (2025). Co-Delivery Evodiamine-Porphyrin Nano-Drug to Enhance Photodynamic-Chemo-Immunotherapy for Triple-Negative Breast Cancer Treatment. Adv. Healthc. Mater..

