# Supplementary material for: Supramolecular Photosensitizers Based on HMeQ[6] and Their Photodynamic Effects on Triple-Negative Breast Cancer Cells
Source: Molecules. 2025 Nov 28;30(23):4576. doi: 10.3390/molecules30234576 (PMC12693310; doi:10.3390/molecules30234576)
Supplement: Supplementary file 1 [file molecules-30-04576-s001.zip › molecules-3967899-supplementary.pdf]

Supramolecular photosensitizers based on HMeQ[6] and their photodynamic effects on 4T1 cells

Beibei Song†, Qingyi Kong†, Bo Xiao*, Ting Huang, Yan Su, Baofei Sun, Guangwei Feng, Xiaojun Wen*, Jian Feng*

Key Laboratory of Human Brain bank for Functions and Diseases of Guizhou Provincial Department of Education, School of Basic Medical Sciences, Guizhou Medical University, Guiyang 550025, China;
* Correspondence: xiaobogzmu@163.com (B.X.); 2547285543@qq.com (X.W.); jfeng@gmc.edu.cn (J.F.)
† These authors contributed equally to this work.

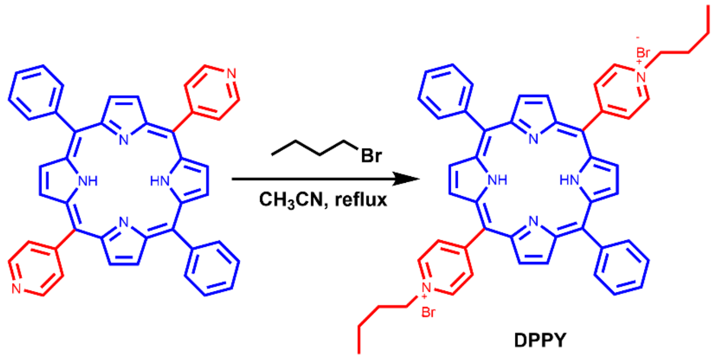

Scheme S1 Synthesis of the guest DPPY

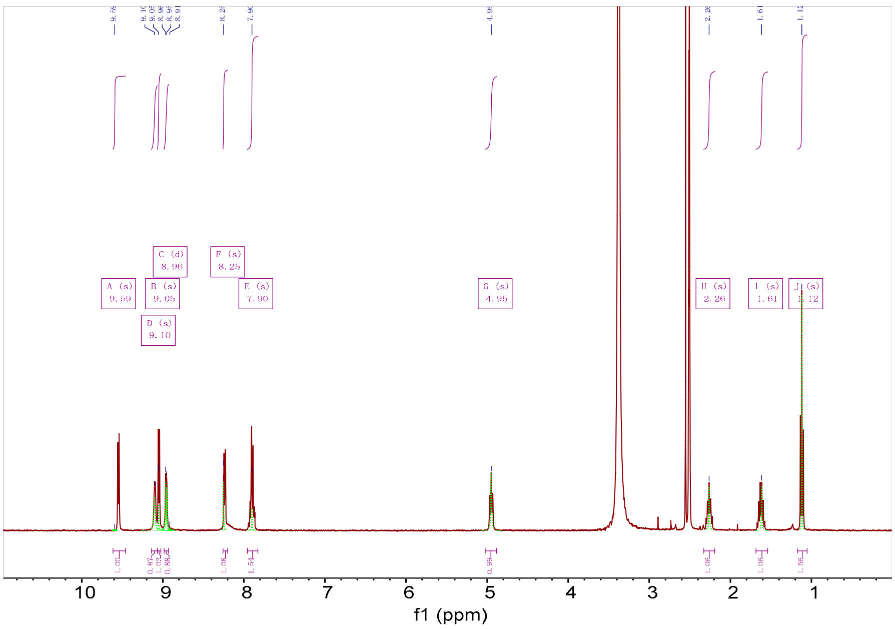

Figure S1 ^{1}H NMR of DPPY (DMSO, 400 MHz, 298 K, 1 mM)

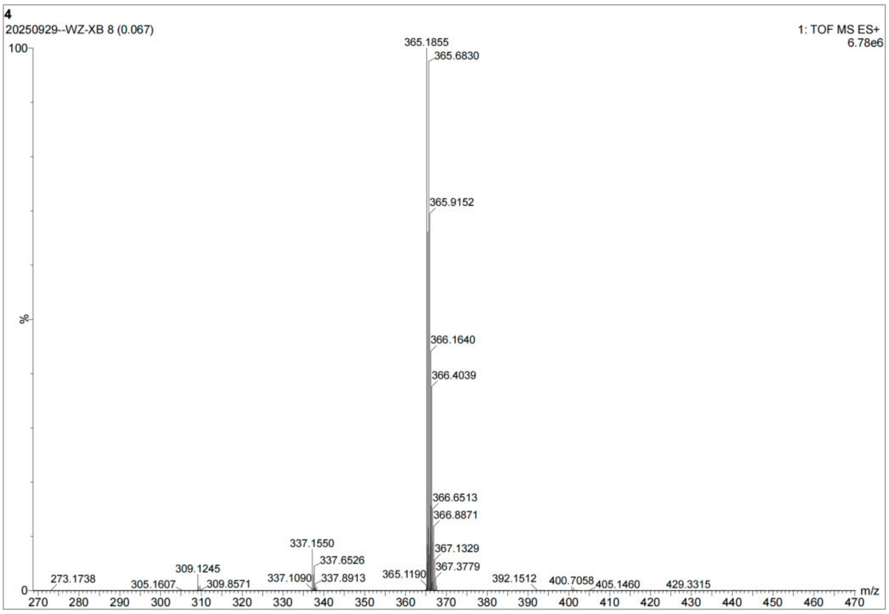

Figure S2 ESI-MS spectrum of DPPY ($[M\text{-}2Br^-]^{2+}$, m/z = 368.1855), (calculated for $[M\text{-}2Br^-]^{2+}$, m/z = 365.1857)

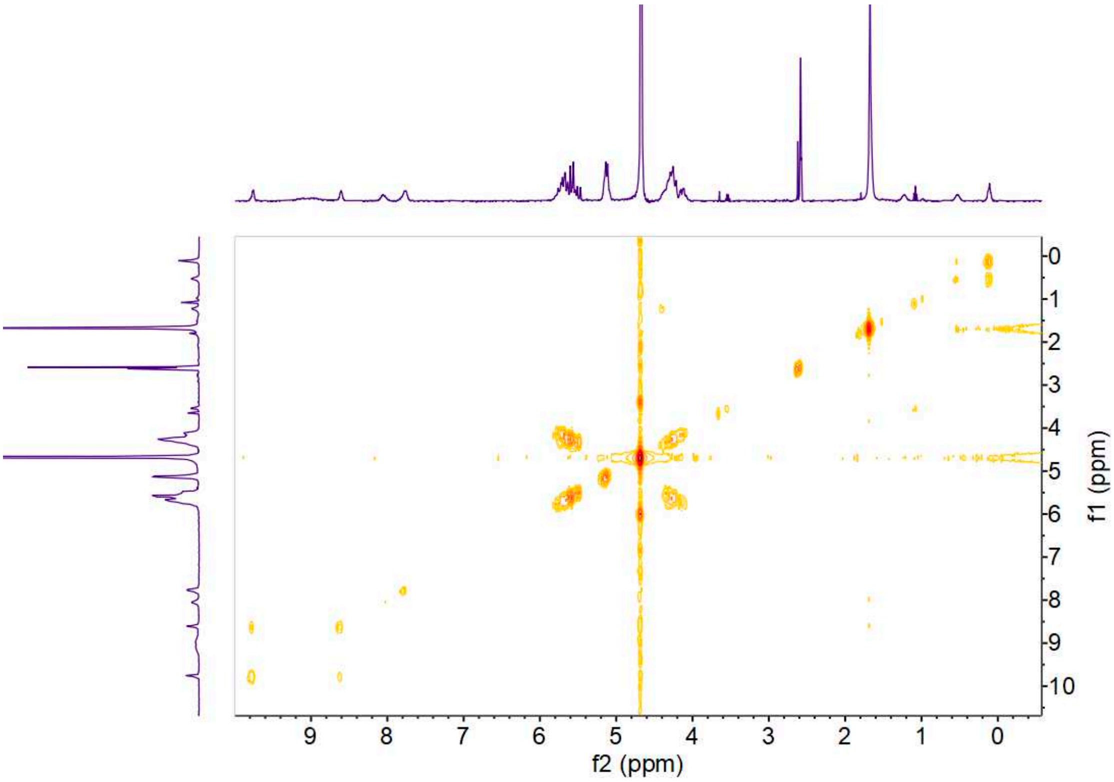

Figure S3 COSY spectrum of DPPY@HMeQ[6] ([DPPY] = 1 mM, [HMeQ[6]] = 2 mM)

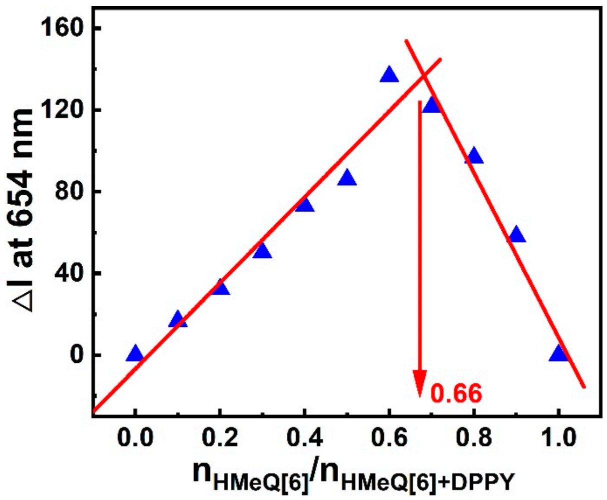

Figure S4 Job's plot of DPPY and HMeQ[6].

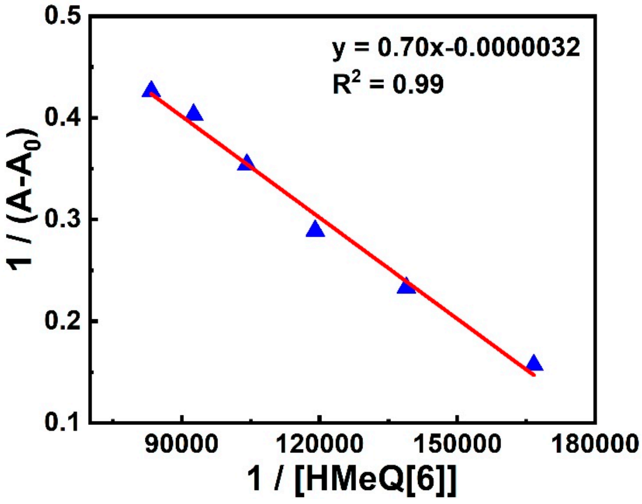

Figure S5 Benesi−Hilderbrand plot of DPPY with HMeQ[6].

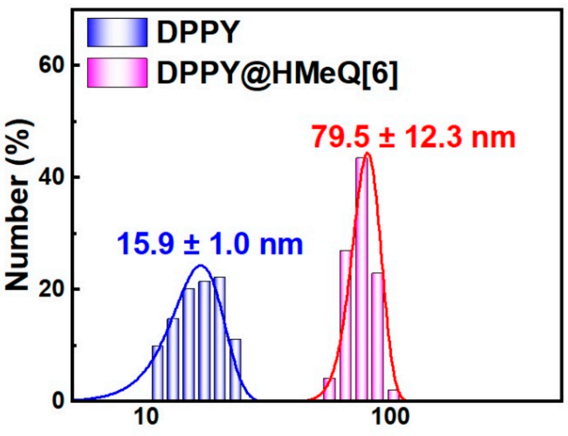

Figure S6 DLS of DPPY and DPPY@HMeQ[6] (pH = 6.52, 20 μM)

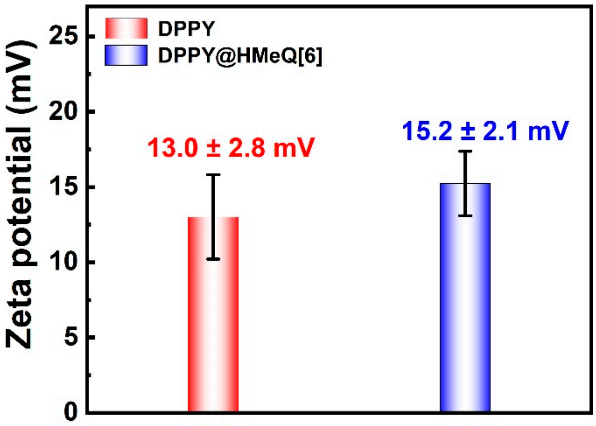

Figure S7 Zeta potentials of DPPY and DPPY@HMeQ[6].

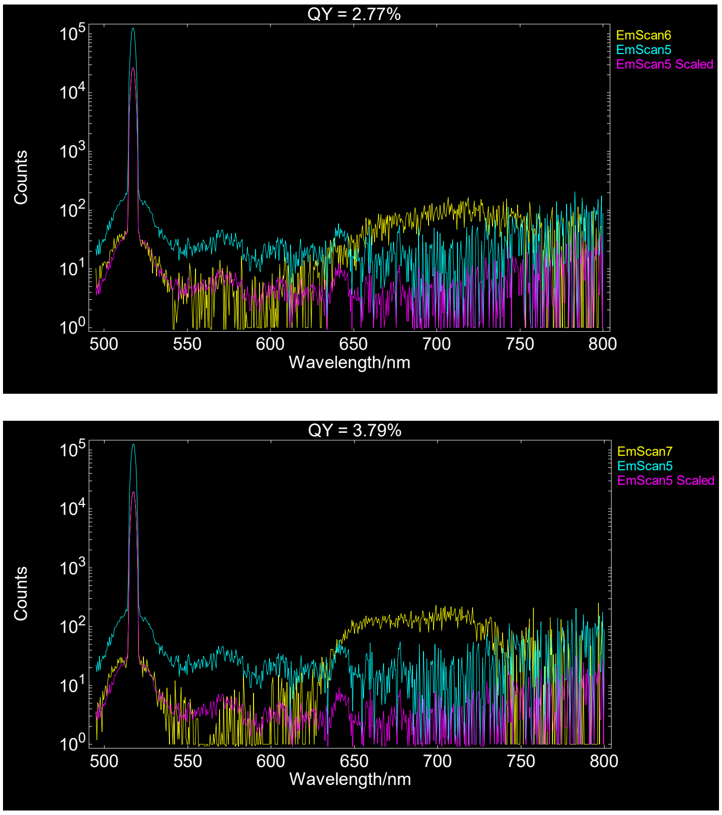

Figure S8 Fluorescence quantum yield of DPPY and DPPY@HMeQ[6] (pH = 6.52, 20 μM)

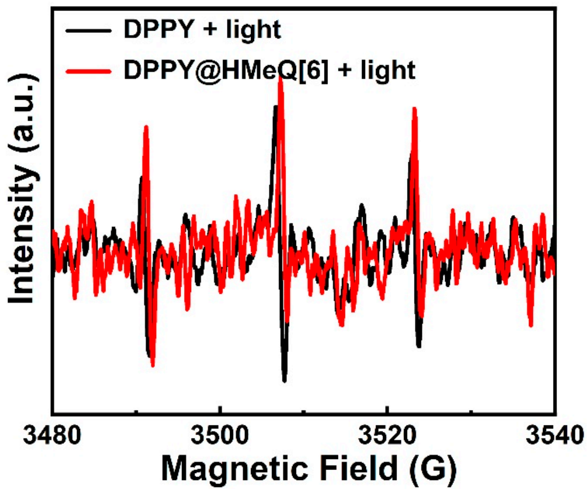

Figure S9 EPR spectra upon irradiation after 10 min of DPPY (black) and DPPY@HMeQ[6] (red).

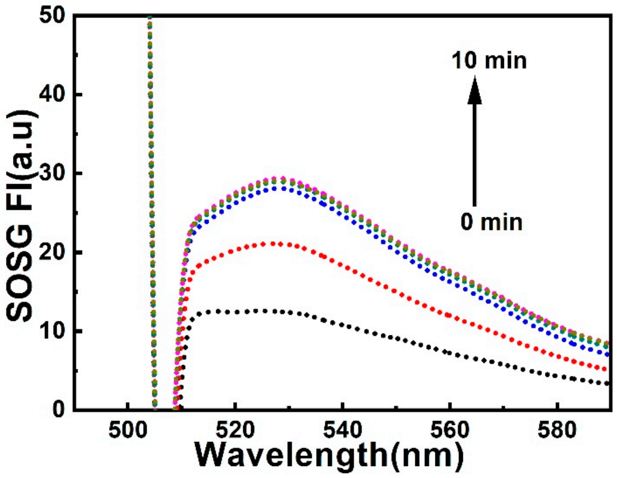

Figure S10 FL intensity of SOSG (5.0 μM) in the presence of DPPY (pH = 6.52, 20 μM) upon irradiation of 10 min.

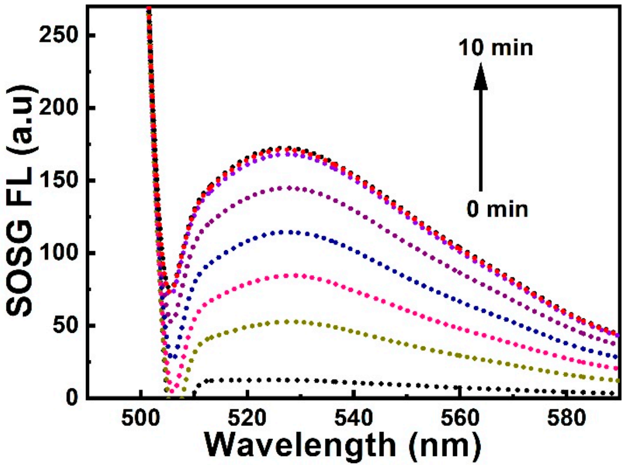

Figure S11 Figure S10 FL intensity of SOSG (5.0 μM) in the presence of DPPY@HMeQ[6] (pH = 6.52, 20 μM) upon irradiation of 10 min.

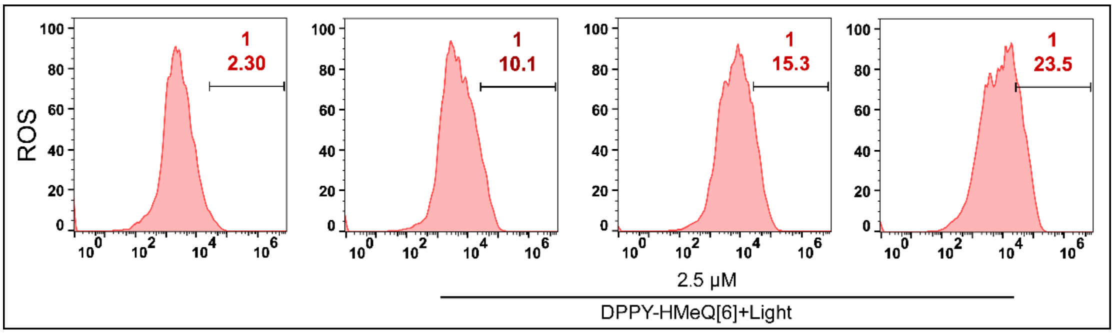

Figure S12 Flow cytometry images of ROS levels obtained through DCFH-DA staining.

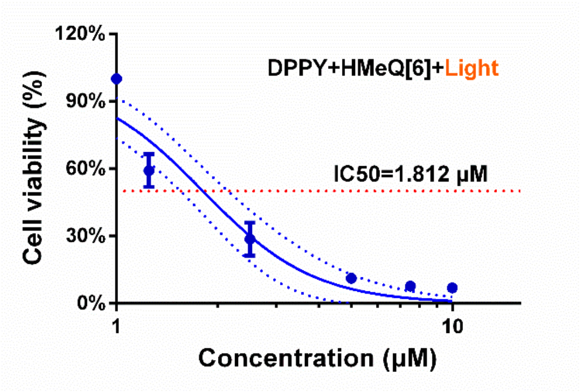

Figure S13 The IC50 of 4T1 cells treated with different doses of DPPY@HMeQ[6] and lighting (n = 3).

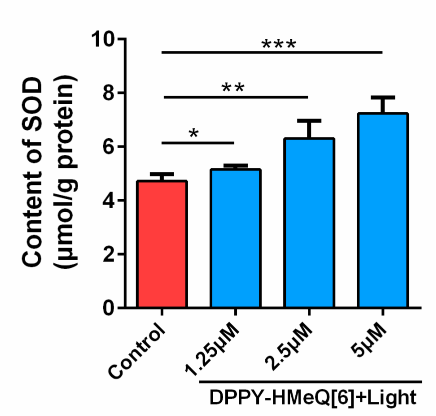

Figure S14 The content of SOD in 4T1 cells after drug intervention (n = 3).

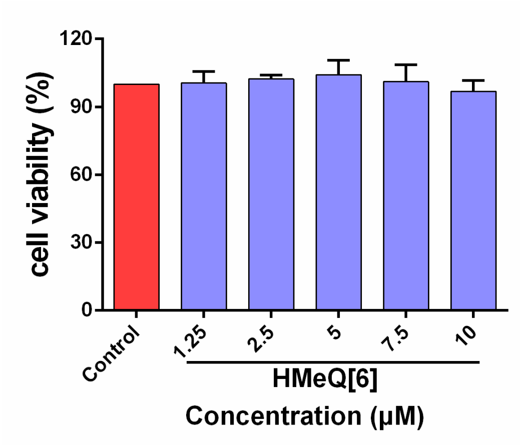

Figure S15 Cell viability of 4T1 cells assessed after incubation with DPPY@HMeQ[6] at 0 to 10 μM for 24h, using the CCK-8 assay (n=3).

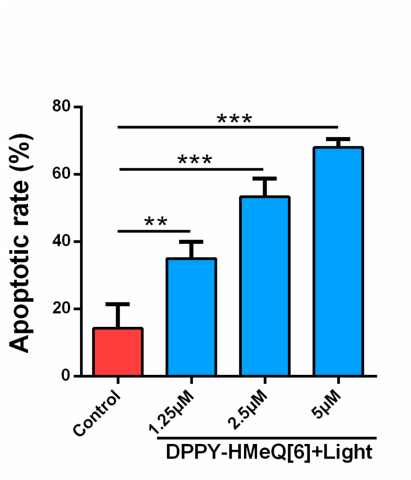

Figure S16 Corresponding statistical analysis of apoptosis (early + late) results (n = 3).
